# A Normal Distribution-Based Methodology for Analysis of Fatal Accidents in Land Hazardous Material Transportation

**DOI:** 10.3390/ijerph15071437

**Published:** 2018-07-07

**Authors:** Cuiping Ren, Qunqi Wu, Chunguo Zhang, Shengzhong Zhang

**Affiliations:** 1 School of Economics and Management, Chang’an University, Xi’an 710064, China; believe.323@163.com (C.R.); wqq@chd.edu.cn (Q.W.); szzhang@chd.edu.cn (S.Z.); 2 Key Laboratory of Road Construction Technology and Equipment, MOE, Chang’an University, Xi’an 710064, China

**Keywords:** hazardous material transportation, fatal accidents, normal distribution, *f*-*N* curve, safety programmes

## Abstract

The deaths of accident occurring in land hazardous material transport (rail and road) is a scale standard for judging accident severity in safety programmes. The *f*-*N* curve is a common practice to express the results from past scattered accident data through curve fitting method, which only estimate the overall trend. For this reason, this paper proposed a simple methodology by combination of normal distribution and *f*-*N* curve. To verify the method, the following three sets of statistical data were selected and analysed in this study: 1932 accidents in over 95 countries (1931–2004) and 322 accidents in China (2000–2008) available in the literature, and 2046 accidents investigated in China (2013–2017). It was found that the mean value curve is almost identical or even better than the best-fitted curve, while the predicted upper and lower limits with 96% reliability (±2σ) covering nearly all the statistical data are beyond the scope of common curve fitting. The result explains the inherent relation between accumulated frequency and deaths in different transport mode, in different country and at different period. This study also provides insights on the evolution of accident severity with the development of social economy and the requirement of safety.

## 1. Introduction

Hazardous material accidents occurring in land transport often result in fateful consequences to the population and environment due to the characteristics of dangerous goods. Programmes on safety improvement have been conducted in many countries, such as the Hazardous material Cooperative Research Program (HMCRP) in the United States, the 5-year plan for safety production in China, and the zero-accident goal by 2050 in EU. Therefore, it is important to evaluate the effects of accident severity in safety programmes and make suitable plans with the development of social economy and the requirement of safety.

Several studies using statistical data have discussed the accident severity with different methods [1,2,3]. For example, Ellis et al. [4] analysed the ratio of hazmat-ship accidents to overall-ship accidents, and Zhang et al. [5] used the proportion of death to injury for hazardous chemical accidents. Abdolhamidzadeh et al. [6] presented the average number of fatalities per accident globally. Brüde [7] described a model for successively monitoring the development of fatalities. These studies mostly make a simple overview of death and injury, without deeply analysis on accident severity. Due to incomplete or inaccurate information of accidents (e.g., economic loss and injured degree) [8,9], it is statistically impossible to conduct a comprehensive study considering all the consequences of hazmat accidents. In this study, only fatal accidents, which are nearly 100% precise, were considered [10].

The *f*-*N* curve (social risk curve) is commonly used to describe the accident severity, by presenting the relationship between the accumulated probability and the number of deaths [11,12,13,14]. Except for marine accidents of hazmat transportation, straight lines are obtained by plotting an f-*N* curve for road, rail and pipeline accidents [15]. The slope of the straight line, obtained by normal curve fitting, is used to evaluate the accident severity as a metric. For example, by comparing the slope of the *f*-*N* curve, Hemmatian et al. [16] and Darbra et al. [17] concluded that hazmat accidents involving domino effects have slightly more severe consequences for the affected population than that of hazmat accidents without domino effects. 

A new method with a mean value curve based on normal distribution was proposed to be identical or even better than the best-fitted curve [18,19]. Furthermore, predictions with 96% reliability (±2σ) of the upper and lower bounds covering nearly all the statistical data are beyond the scope of normal curve fitting [18], and this may also be used for the prediction of severity analysis on fatal accidents. On this basis, the aim of this paper is to propose a methodology based on normal distribution and the *f*-*N* curve and, therefore, to explore ways to decrease fatal accidents with the related safety policies and programmes. 

## 2. Methodology

### 2.1. Data Resource

Hazardous material accidents mostly happened during transportation, especially in land transport; in China, at least 80% of hazmat transportation accidents occurred on the road [2]. However, the accident statistical data is limited due to underreported accidents—for example, some states only report accidents that result in property damage above a specific threshold dollar amount, while others require the degree of vehicle damage to be above a certain level [20]. In addition, it is well-known that individuals involved in accidents involving no injury or minor injury are far less likely to have their crashes reported to the police. A technical report by the National Traffic Safety Administration [21] estimated that 25% of minor injury accidents and half of no-injury crashes are unreported—a sharp contrast to fatal crashes, for which the reporting rate is nearly 100% [10]. 

Recognizing the limited accident information is an important consideration in selecting an appropriate sample data. Hence, this study selected accidents with at least one death that occurred in land hazardous material transportation, and three sets of data were analysed in this study. One data set is digitized from Oggero conducting a survey of 1932 accidents (including 242 fatal accidents) occurred during the land transport of hazardous material from over 95 countries. Oggero et al. [11] analysed fatalities with an *f*-*N* curve which provides a comparative study for testing the normal distribution-based method. The second sample data set is digitized from Yang et al. [12] with 322 accidents occurred during the road hazmat transportation in China from 2000 to 2008. The reliability of graphic digitalization has been checked by comparing with the original result in the literature. It is found that the digitized error is no more than 1%, which is acceptable. In addition, deaths analysis with an *f*-*N* curve was also carried out in Yang et al. [12]. The third data set contains 2046 accidents (including 217 accidents caused deaths) happened in road hazardous material transportation in China from 2013 to 2017, and these data are investigated from the Journal of Safety and Environment and Chemical Accidents Information Network. Detailed information was gathered from the newspaper and the Internet. 

### 2.2. Method

The *f*-*N* curve is a method expressing the results of risk evaluation. It refers to accumulated frequency of N people or more than N people affected in accidents. For evaluating the severity of fatal accidents in hazmat transportation, the most commonly used method is to plot *f*-*N* curve by showing the relation between accumulated frequency and number of deaths. Accidents with at least one fatality were selected and grouped according to the number of deaths. The cumulative probability or frequency is calculated by the following expression:

(1) $$P_{\left(x\geq N\right)}=F_{j}=\frac{\sum_{i=j}^{n}N_{i}}{\sum_{i=1}^{n}N_{i}}$$

where *N* is the number of deaths (*x*-axis), $P_{\left(x\geq N\right)}=F_{j}$ is the probability that the number of deaths is equal or more than *N* (*y*-axis) in an accident, *n* is the total number of categories or rankings, and *N_i_* is the number of accidents in a given category *i*.

In a log-log system, *F_j_* is roughly linear to *N_i_* for land transport accidents ($\mathrm{lg}F_{j}=b\cdot \mathrm{lg}N_{i}$), which is different to the marine transport that has a hump in the *f*-*N* curve [15]. To better present this distribution in quantification and visualization, previous studies have obtained the best fitted curve with a slope of *b* [11,12,13,14]. The slope of *b* refers to relative probability, which means, for example, that the probability of an accident involving 10 or more deaths is 10^−*b*^ ($F=10^{b}/100^{b}=10^{-b}=N^{-b}$, when *N* = 10) times greater than that of an accident involving 100 or more deaths. However, the value of *b* is different in various conditions—for example, the slope is −0.81 from Oggero et al. [11] while −0.84 was obtained in Vílchez [14]. Therefore, it is essential to obtain the value of *b* in precise.

The slope of *b* is defined by the intersection of two criteria—*F* and *N*, and is normally distributed by using Equation (2). Therefore, the distribution of *b_i_* can be obtained with the corresponding mean value *μ* and the standard deviation *σ*.

(2) $$f(x)=\frac{1}{\sigma \sqrt{2\pi }}e^{-\frac{{\left(x-\mu \right)}^{2}}{2\sigma^{2}}}$$

The distribution of *b_i_*, closest to a normal distribution with the smallest standard deviation is used to determine the relative probability and make a prediction of an *f*-*N* curve with the mean (*μ*) and deviation (*σ*). With the accumulated probability (*F*), the number of deaths (*N_i_*), and the corresponding standard deviations (*σ*), the equation of a straight line can be written to include the mean value curve and upper and lower limits with 96% reliability (±2*σ*), i.e.,

(3) $$F=\left\{ \begin{array}{l} (\bar{b}+2\sigma )\times N_{i}\\ \bar{b}\times N_{i}\\ (\bar{b}-2\sigma )\times N_{i} \end{array}\right.$$

The above equation means that the relative probability can be fully predicted with the mean *μ* ($\mu =\bar{b}$) and the standard deviation *σ*. The severity of accidents can then be analysed with an accurate mean value curve and upper and lower limits that address the fluctuation of accidents in hazardous material transportation.

To show the usefulness of the methodology, we examine the relation between accumulated frequency and number of deaths in different transport mode, in different country and at different period. 

## 3. Results

### 3.1. Severity Analysis of Fatalities in Different Transport Mode

The severity of fatal accidents occurred in different transport mode is different. In this paper, by plotting an *f*-*N* curve based on normal distribution, we conducted a comparative study to analyse the severity of fatalities of road and rail transport accidents. There are 31 results (*F*, *N_i_*) in road and 18 results (*F*, *N_i_*) in rail both digitized from Oggero et al. [11], listed in Table 1 and Table 2, respectively. In the table, NA means *b_i_* is not available in a log-log system.

**Figure 1:**
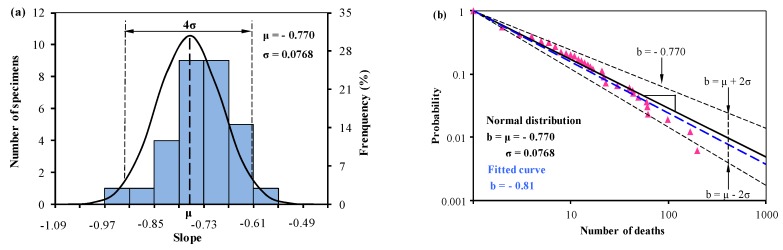
(**a**) Normal distribution analysis of the slope *b_i_* determining the relation between the accumulated frequency and number of deaths, and (**b**) predictions from Equations (1) and (3) based on the normal distribution analysis of *b_i_* in (**a**) together with the statistical data in road accidents from 1931 to 2004 digitized from Oggero et al. [11].

Based on the corresponding experimental data in Oggero et al. [11], the slope of *b_i_* is normally distributed from Equation (2) with the mean value μ = −0.770 and the standard deviation σ = 0.0768 (in Figure 1a). The *f*-*N* curves of the mean value and the predictions of upper and lower limits from Equation (3) were plotted in Figure 1b, together with previous result—a fitted curve (in blue colour). The fitted curve is plotted from the digitized data and is identical to the original result in the literature.

The mean value curve (the black straight line) in Figure 1b, based on normal distribution, is slightly different from the fitted curve (the blue dotted line). The mean value curve (*b* = −0.77) describes these experimental results even better than the best-fitted curve (*b* = −0.81) [11]. The same result that the normal distribution is identical or better than the curve fitting was published by Zhang et al. [18]. The gradient of the upper dashed line is −0.616, and the gradient of the lower dashed line is −0.924. This indicates that the relative frequency of an accident involving 10 or more deaths versus an accident involving 100 or more deaths, ranges from 4.1 to 8.4 (*F*= 10^−*b*^), and the mean value is 5.9.

**Figure 2:**
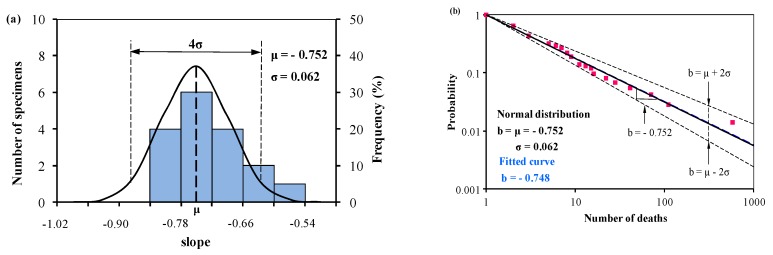
(**a**) Normal distribution analysis of the slope *b_i_* determining the relation between the accumulated frequency and number of deaths, and (**b**) predictions from Equation (1) and Equation (3) based on the normal distribution analysis of *b_i_* in (**a**) together with the statistical data in rail accidents from 1931 to 2004 available in Oggero et al. [11].

Table 2 presents the accumulated frequency (*F*), the number of deaths (***N_i_***) and the slope of *b_i_* for rail transport accidents (NA means *b_i_* is not available). The same as road accidents, the slope of *b_i_* in rail accidents is normally distributed from Equation (2) with the mean value μ = −0.752 (approximately 0.75 in the literature) and the standard deviation σ = 0.062. Figure 2 shows the distribution of fatalities and the *f*-*N* curves with the mean value and the predictions of upper and lower limits from Equation (3), together with a fitted curve from Oggero et al. [11] on a log-log system.

In Figure 2, the slope of the fitted curve (−0.748) [11] is almost identical to the slope of the mean value curve (0.752). *b_i_* ranges from −0.873 to −0.630 using Equation (3). This means, for example, an accident involving 10 or more deaths is 4.3 to 7.5 (*F*= 10^−*b*^) times greater than an accident involving 100 or more deaths, and the mean value is 5.6.

Results show that the slope of *f*-*N* curve (the mean value curve) of rail accidents is less than the slope of road accidents, showing fatal accidents occurred in rail transport is more severe than in road transport in general. However, the scope of predictions with upper and lower bounds in road accidents (−0.924–−0.616) contains the scope of rail accidents (−0.873–−0.630), and this indicates that the severity of fatal accidents occurred in road transport is more severe in the upper bound and is less severe in the lower bound than that of rail transport. 

### 3.2. Severity Analysis of Fatalities in Different Country

The severity of fatal accidents is closely pertinent to the development of the country. Oggero et al. [11] analysed the fatalities of accidents by grouping countries into three groups: (1) United States, Canada, Australia, Japan, New Zealand and Norway; (2) the European Union; and (3) the rest of the world. Countries in Group 1 and Group 2 are all well-developed, while countries in Group 3 are almost developing countries. Oggero et al. [11] concluded that the severity of accidents in the first two groups is similar and in group (3) the severity of accidents is clearly higher than in the first two. 

To conduct a comparative study on the differences of severities influenced by the level of development in different countries, we use the same sample and groups from Oggero’s [11]. In total, 13 data points (*F*, *N_i_*) of Group 1, 11 data points (*F*, *N_i_*) of Group 2, and 33 data points (*F*, *N_i_*) of Group 3 were digitized, and listed in Table 3. All these data contains fatal accidents occurred in both road and rail hazmat transportation in the same period. NA means *b_i_* is not available in a log-log system in Table 3.

**Figure 3:**
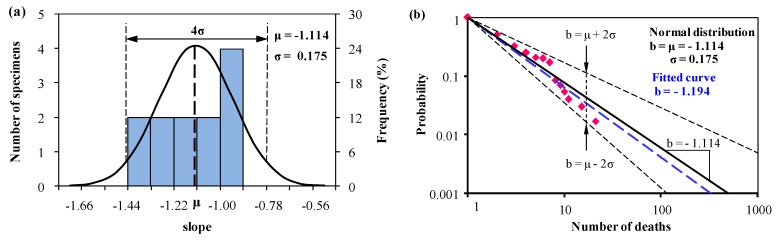
(**a**) Normal distribution analysis of the slope *b_i_*, and (**b**) predictions from Equation (1) and Equation (3) based on the normal distribution analysis of *b_i_* in (**a**) for Group 1 together with the statistical data (1931–2004) available in Oggero et al. [11].

**Figure 4:**
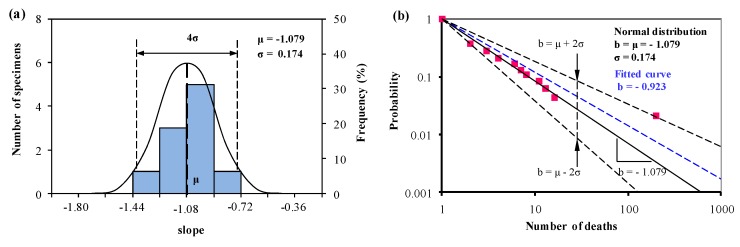
(**a**) Normal distribution analysis of the slope *b_i_*, and (**b**) predictions from Equation (1) and Equation (3) based on the normal distribution analysis of *b_i_* in (**a**) for Group 2 together with the statistical data (1931–2004) available in Oggero et al. [11].

**Figure 5:**
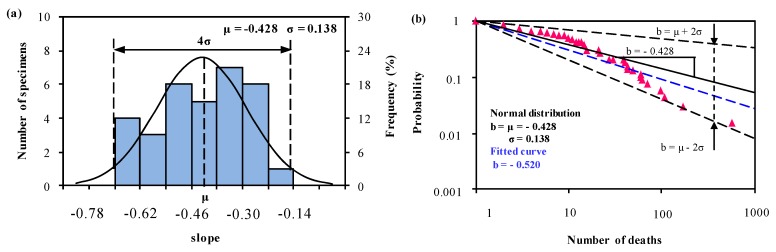
(**a**) Normal distribution analysis of the slope *b_i_*, and (**b**) predictions from Equation (1) and Equation (3) based on the normal distribution analysis of *b_i_* in (**a**) for Group 3 together with the statistical data (1931–2004) available in Oggero et al. [11].

According to Equation (2), we draw the same conclusion that the slope of *b_i_* is normally distributed and equal to the original literature for all the three groups. Based on a normal distribution, we obtain the mean value curve and the predictions with upper and lower lines, together with the fitted curve commonly used, and they are listed in Figure 3, Figure 4 and Figure 5.

In Figure 3 and Figure 4, the mean value of Group 1 (*b* = −1.114) is very close to the mean value in Group 2 (*b* = −1.079), and this two groups include most developed countries. This indicates that the severity of fatalities occurred in developed countries is similar. While the predicted slopes with the upper and lower bounds describe the gap between Group 1 and Group 2 by using Equation (3). In Group 1, the relative probability for the deaths ranges from 5.8 to 29.1, and in Group 2, this value ranges from 5.4 to 26.7. This indicates in the countries (i.e., United States, Canada, Australia, et al.) of Group 1, fatalities caused in the accident of hazardous material transportation may appear to higher volatility than in EU of Group 2.

In Group 3, the slope of the mean value curve is −0.428, and is much higher than the first two groups. The slopes of predictions with upper and lower bounds ranges from −0.152 to −0.704 (Figure 5b), and this means an accident involving 10 or more deaths is 1.4 to 5.1 (*F* = 10^−*b*^) times greater than an accident involving 100 or more deaths. Comparing to the first two groups, the severity of fatalities in Group 3 is the highest, and this result is in good agreement with the study published by Oggero et al. [11].

### 3.3. Severity Analysis of Fatalities at Different Period

To provide an updated survey and conduct a comparative study on the severity of fatal accidents occurred in the road transportation of hazardous material, this study selected 322 accidents (2000–2008) with 8 data points (*F*, *N_i_*) digitized from Yang et al. [12] and investigated 2046 accidents including 217 fatal accidents (2013–2017) with 11 data points (*F*, *N_i_*) in China. All the data points are listed in Table 4 and NA is not available.

**Figure 6:**
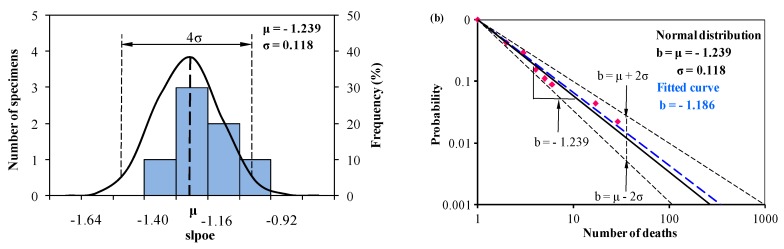
(**a**) Normal distribution analysis of the slope *b_i_* determining the relation between the accumulated frequency and number of deaths, and (**b**) predictions from Equation (1) and Equation (3) based on the normal distribution analysis of *b_i_* in (**a**) together with the statistical data from 2000 to 2008 in China digitized from Yang et al. [12].

**Figure 7:**
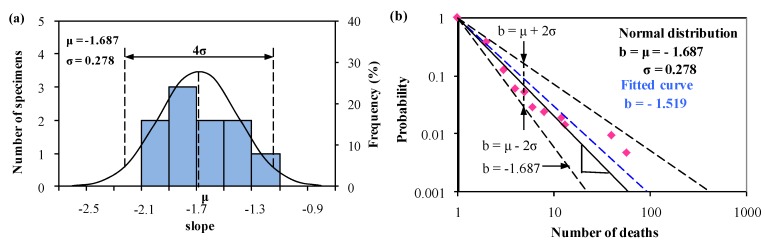
(**a**) Normal distribution analysis of the slope *bi* determining the relation between the accumulated frequency and number of deaths, and (**b**) predictions from Equation (1) and Equation (3) based on the normal distribution analysis of *b_i_* in (**a**) together with the statistical data from 2013 to 2017 in China.

As shown in Table 4, it is easy to establish a normal distribution for the slope *b_i_* with specified or 96% reliability. The normal distribution analysis on slope *b_i_* based on Equation (2), and the predictions with mean value and with upper and lower bounds based on Equation (3), are shown in Figure 6 and Figure 7, together with the corresponding best-fitted curves.

Figure 6a shows that *b_i_* is normally distributed with the mean value μ = −1.239 and the standard deviation σ = 0.118. The fitted-curve (*b* = −1.186) plotted by using the digitized data is slightly different from the original result in the literature. This small error may come from digitization of the experimental data. However, the digitized error is acceptable. In Figure 6b, different from the fitted curve, the mean value curve is close to the first few data points with less than or equal to 6 deaths, which is more representative. 

Figure 7a shows that *b_i_* is normally distributed with the mean value μ = −1.687 and the standard deviation σ = 0.278. In Figure 7b, the mean value curve is lower than the best-fitted curve because accidents are within severe fluctuation (i.e., 40 and 58 deaths in accidents) in this sample. This indicates the mean value curve is better than the fitted curve.

Figure 6 and Figure 7 show that from 2000–2008, the slope of the mean value curve (*μ* = −1.239) is much higher than that in 2013–2017 *(μ* = −1.687). Moreover, the slope of the predicted upper and lower bounds with 96% reliability ranges from −1.475 to −1.003 in 2000–2008, and from −2.232 to −1.142 in 2013–2017. This indicates China has made great improvement in decreasing the deaths of accident occurred in road hazardous material transportation.

## 4. Discussion

### 4.1. Fatal Transportation Accidents by Road and Rail

According to the statistical data of hazardous material transportation, accidents occurring on a railway are much less frequent than that on a road, while research shows that the consequence of an accident is likely to be more severe if the accident occurs on a railway rather than on a road [11]. The same conclusion was obtained through the analysis of the mean value curve—the slope is −0.77 for road accidents (Figure 1) and −0.75 for rail accidents (Figure 2). However, predictions on the upper and lower bounds with 96% reliability (±2σ) show that the relative probability of the fatalities of road accidents (4.1 to 8.4) ranges larger than the rail accidents (4.3 to 7.5). This indicates that in some cases, the road accident-severity may be more severe than rail because the consequences of an accident are determined by possible causes, for example, impact failure, mechanical failure, human factor and external conditions, and by emergency responses. Besides, railway hazmat transportation has a fixed route system and each train involves multiple cars with hazardous material. Once a hazmat train derailed, multiple tank cars may release [22]. This usually leads to severe consequences in a particular scope. Research on the train derailment severity of derailed cars has been carried out in recent years [23,24,25,26].

### 4.2. The Impact of the Development Levels

In Group 1 and Group 2, the slopes of the mean value curves are approximately −1.1 (Figure 3, Figure 4 and Figure 5), which is in agreement with the result in Oggero et al. [11] and Carol et al. [27]. Subtle differences can be described by using the upper and lower boundaries with 96% reliability. In Group 1, the relative probability ranges from 5.8 to 29.1, while in Group 2 this value ranges from 5.4 to 26.7. This indicates that the consequences of an accident are likely to be identical or more severe if the accident occurs in the European Union than in the countries of Group 1. In Group 3, the relative probability of upper and lower limits ranges from 1.4 to 5.1, which is far less than the first two groups. This result is identical to the hypothesis of Law et al. (2011) [28]. Considering the levels of economic development, it is found that the developing countries in Group 3 are at the early stage of industrialization and pay little attention on safety programmes. In developed countries, the severity of fatal accidents declines as the per capita income increases and the improvement in safety management.

### 4.3. The Evolution of Accident Severity

Combining the five stages in safety production and the development of the social economy [1,29], the severity of fatal accidents occurred in hazmat transportation will undergo five stages correspondingly. The five stages are as follows: Stage 1—the severity is relatively low with few transportation accidents in agricultural economy; Stage 2—the severity increases as the number of accidents increase in early industrialization; Stage 3—the severity reaches a general trend with fluctuations in middle industrialization; Stage 4—a general decline of accident severity in advanced industrialization is seen; Stage 5—a stabilized period for accident severity in the information society is seen.

By comparing the slope of the mean value curve in Figure 6b and in Figure 7b, it shows that the consequences of an accident are likely to be slightly less severe if the accident occurred in 2013–2017 rather than in 2000–2008. This indicates that in China the severity of fatal accidents in hazardous material transportation has decreased as a whole. Considering the above result and the reduction of safety accidents [30,31,32], China is at Stage 4 in the evolution of the accident severity in hazardous material transportation. This achievement may be attributed to the development of social economy and the implementation of national safety programmes, such as the 5-year plans for safety production.

## 5. Conclusions

For the fatalities analysis of accidents, the common practice is to fit the average results (i.e., fitting an *f*-*N* curve) regardless how large the scatters in statistical results are. In this study, by combination of normal distribution and the *f*-*N* curve, a simple and reliable methodology is proposed for accident data analysis. On the basis of this theory, we presented the fatal accidents by groups of transport modes (i.e., road and rail), countries, and periods, respectively. It is evident that the mean value curves are almost identical or even better than the linear-fitted curves, but the predicted upper and lower limits with 96% reliability covering nearly all the statistical data are beyond the scope of common curve fitting. With the development of social economy and safety requirements, the evolution of accident-severity undergoes five stages. Usually in developed countries the severity of fatalities is less severe than that in developing countries, because infrastructures and safety measures during the transportation of hazardous material are not effective in developing countries.

In principle, the methodology presented in this study on the fatalities analysis can be used to evaluate safety policies and propose countermeasures for the transportation of hazardous material. This will make relevant study more objective than the common practice of curve fitting as routinely done.

## Figures and Tables

**Table 1:** Accumulated frequency of road accidents with *N* deaths digitized from Oggero et al. [11].

*N_i_*	*F*	*b_i_*	*N_i_*	*F*	*b_i_*	*N_i_*	*F*	*b_i_*
1	1.000	NA	12	0.170	−0.713	40	0.060	−0.763
2	0.550	−0.862	13	0.160	−0.714	43	0.054	−0.776
3	0.420	−0.790	14	0.150	−0.719	44	0.049	−0.797
4	0.390	−0.679	15	0.140	−0.726	50	0.042	−0.810
5	0.330	−0.689	16	0.130	−0.736	60	0.036	−0.812
6	0.310	−0.654	17	0.120	−0.713	61	0.030	−0.853
7	0.270	−0.673	21	0.110	−0.714	62	0.023	−0.914
8	0.220	−0.728	22	0.090	−0.719	100	0.019	−0.861
9	0.210	−0.710	23	0.071	−0.726	170	0.012	−0.861
10	0.200	−0.699	30	0.064	−0.736	200	0.006	−0.966
11	0.190	−0.693						
Normal distribution: *μ* = −0.770, *σ* = 0.0768								

**Table 2:** Accumulated frequency of rail accidents with *N* deaths digitized from Oggero et al. [11].

*N_i_*	*F*	*b_i_*	*N_i_*	*F*	*b_i_*
1	1000	NA	13	0.130	−0.795
2	0.650	−0.621	15	0.120	−0.783
3	0.430	−0.768	16	0.095	−0.849
5	0.320	−0.708	22	0.081	−0.813
6	0.290	−0.691	28	0.067	−0.811
7	0.270	−0.673	41	0.054	−0.786
8	0.220	−0.728	71	0.042	−0.744
9	0.190	−0.756	110	0.028	−0.761
11	0.140	−0.820	581	0.014	−0.671
Normal distribution: *μ* = −0.752, *σ* = 0.062					

**Table 3:** Accumulated frequency of land transport accidents with *N* deaths for the three groups digitized from Oggero et al. [11].

**Group 1: US, Canada, Australia, etc.**					**Group 2: EU**				
***N_i_***	***F***		***b_i_***		***N_i_***		***F***		***b_i_***
1	1.000		NA		1		1.000		NA
2	0.520		−0.943		2		0.370		−1.434
3	0.330		−1.009		3		0.280		−1.159
4	0.250		−1.000		4		0.210		−1.126
5	0.210		−0.970		6		0.170		−0.989
6	0.200		−0.898		7		0.130		−1.048
7	0.170		−0.911		8		0.110		−1.061
8	0.085		−1.185		11		0.084		−1.033
9	0.070		−1.210		13		0.064		−1.072
10	0.054		−1.268		16		0.043		−1.135
11	0.040		−1.342		200		0.021		−0.729
15	0.030		−1.295						
21	0.017		−1.338						
Normal distribution: *μ* = −1.114, *σ* = 0.175						Normal distribution: *μ* = −1.079, *σ* = 0.174			
**Group 3: the Rest of the World**									
***N_i_***	***F***	***b_i_***		***N_i_***	***F***	***b_i_***	***N_i_***	***F***	***b_i_***
1	1.000	NA		12	0.430	−0.340	43	0.160	−0.487
2	0.880	−0.184		13	0.410	−0.348	44	0.140	−0.520
3	0.740	−0.274		14	0.410	−0.338	50	0.130	−0.522
4	0.650	−0.311		15	0.370	−0.367	60	0.110	−0.539
5	0.640	−0.277		16	0.310	−0.422	61	0.100	−0.560
6	0.610	−0.276		21	0.310	−0.385	62	0.088	−0.589
7	0.580	−0.280		22	0.270	−0.424	71	0.075	−0.608
8	0.550	−0.287		28	0.210	−0.468	100	0.057	−0.622
9	0.540	−0.280		30	0.210	−0.459	110	0.043	−0.669
10	0.490	−0.310		40	0.200	−0.436	170	0.030	−0.683
11	0.480	−0.306		41	0.180	−0.462	581	0.015	−0.660
Normal distribution: *μ* = −0.428, *σ* = 0.138									

**Table 4:** Accumulated frequency of hazardous material accidents occurred in road transportation with *N* deaths from Yang et al. [12] and investigation in different periods of China.

2000–2008 (Yang et al. [12])			2013–2017 (Investigated Data)		
***N_i_***	***F***	***b_i_***	***N_i_***	***F***	***b_i_***
1	1.000	NA	1	1.000	NA
2	0.423	−1.241	2	0.378	−1.404
3	0.288	−1.132	3	0.124	−1.897
4	0.154	−1.350	4	0.060	−2.031
5	0.110	−1.371	5	0.051	−1.853
6	0.089	−1.351	6	0.028	−2.003
17	0.044	−1.101	8	0.023	−1.813
29	0.022	−1.133	12	0.018	−1.607
			13	0.014	−1.669
			40	0.009	−1.271
			58	0.005	−1.325
Normal distribution: *μ* = −1.239, *σ* = 0.118			Normal distribution: *μ* = −1.687, *σ* = 0.278		
